# Investigating the role and regulation of GPNMB in progranulin-deficient macrophages

**DOI:** 10.3389/fimmu.2024.1417836

**Published:** 2024-09-26

**Authors:** Drew A. Gillett, Noelle K. Neighbarger, Cassandra Cole, Rebecca L. Wallings, Malú Gámez Tansey

**Affiliations:** ^1^ Center for Translational Research in Neurodegenerative Disease, University of Florida, Gainesville, FL, United States; ^2^ Department of Neuroscience, University of Florida, Gainesville, FL, United States; ^3^ McKnight Brain Institute, University of Florida, Gainesville, FL, United States; ^4^ Norman Fixel Institute for Neurological Diseases, Gainesville, FL, United States

**Keywords:** progranulin, GPNMB, MITF, macrophage, inflammation

## Abstract

**Introduction:**

Progranulin (PGRN) is a holoprotein that is internalized and taken to the lysosome where it is processed to individual granulins (GRNs). PGRN is critical for successful aging, and insufficient levels of PGRN are associated with increased risk for developing neurodegenerative diseases like AD, PD, and FTD. A unifying feature among these diseases is dysregulation of peripheral immune cell populations. However, considerable gaps exist in our understanding of the function(s) of PGRN/GRNs in immune cells and their role in regulating central-peripheral neuroimmune crosstalk. One of the most upregulated genes and proteins in humans with GRN haploinsufficiency and in aged *Grn* knock-out (KO) mice is glycoprotein non-metastatic B (GPNMB) but its normal role within the context of immune crosstalk has not been elucidated.

**Methods:**

To address this gap, peritoneal macrophages (pMacs) from 5-to-6-month old WT and *Grn* KO mice were assessed for Gpnmb expression and stimulation-dependent cytokine release in the presence or absence of the Gpnmb extracellular domain (ECD). Cellular localization, as well as inhibition of, the microphthalmia-associated transcription factor (MITF) was assessed to determine its mechanistic role in Gpnmb overexpression in *Grn* KO pMacs.

**Results:**

We observed an increase in GPNMB protein and mRNA as a result of insufficient progranulin in peripheral immune cells at a very early age relative to previous reports on the brain. Stimulation-dependent cytokine release was decreased in the media of *Grn* KO pMacs relative to WT controls; a phenotype that could be mimicked in WT pMacs with the addition og GPNMB ECD. We also found that MITF is dysregulated in *Grn* KO pMacs; however, its nuclear translocation and activity are not required to rescue the immune dysregulation of *Grn* KO macrophages, suggesting redundancy in the system.

**Discussion:**

These findings highlight the fact that knowledge of early-stage disease mechanism(s) in peripheral populations may inform treatment strategies to delay disease progression at an early, prodromal timepoint prior to development of neuroinflammation and CNS pathology.

## 1 Introduction

Progranulin is a glycosylated holoprotein that is composed of seven and a half cysteine-enriched repeats, called granulins, that are distributed as “beads on a string” with short linker regions joining the repeats together (1). Nascent progranulin is translated into the endoplasmic reticulum (ER), where ER-resident chaperone and folding proteins aid in the cysteine-cysteine disulfide bonds that permit the beta-fold structure of the granulins. After glycosylation, progranulin can be secreted out of the cell or intracellularly trafficked to the endo-lysosomal pathway directly. Extracellular progranulin enters the cell via interaction with its receptor, sortilin (Sort1) (2), or by piggybacking onto a similar holoprotein, prosaposin, which is internalized with LRP1 and M6PR (3). Secreted progranulin circulates throughout the body fluids, including blood serum and cerebrospinal fluid (CSF) as a dimer (4). In this way, the level of progranulin expression is spread throughout the body, allowing even low-producing cells to take up sufficient amounts of progranulin.

Progranulin is critical for successful aging (5). Multiple mechanisms can influence progranulin expression, but loss of full progranulin expression, approximately 96-125ng per mL of blood serum (6, 7), can increase risk of disease. The silencing of one *GRN* allele is associated with an increased risk for Frontotemporal Dementia (FTD), but the silencing of both alleles results in Neuronal Ceroid Lipofuscinosis (NCL-11) (8, 9). Furthermore, other age-related neurodegenerative diseases, like Parkinson’s Disease (PD) and Alzheimer’s Disease (AD), are associated with decreased progranulin levels (10–18), suggesting common underlying mechanisms regarding the role of progranulin in healthy aging.

The specific functions of progranulin are multi-faceted and impact specific cell populations differently. Peripheral immune cells rely on progranulin. Clinically symptomatic *GRN*-mutation carriers showed increased soluble Cluster of Differentiation 163 (CD163) and CC Chemokine Ligand 18 (CCL18) serum levels relative to healthy controls, suggesting increased peripheral macrophage activity (19). In addition, lipopolysaccharide binding protein (LBP) levels correlated with white-matter changes in the frontal lobe and Clinical Dementia Rating (CDR)-FTLD sum of boxes (SB) scores of clinically symptomatic *GRN*-FTD patients, indicating that peripheral immune activity correlates with region-specific brain changes and clinically relevant behavior. When macrophages isolated from progranulin-deficient mice were challenged with lipopolysaccharide (LPS), they showed increased transcription of *Mcp-1*, *Il-12p40*, and *Tnf*, as well as decreased *Il-10* transcription, relative to wild-type (WT) controls (20).

In the brain, progranulin is associated with neuroprotection. The precise mechanism(s) are unclear, but progranulin modulates neuroinflammation and reduces brain volume loss in stroke and head injury paradigms (21, 22) and also prevented dopaminergic neuron loss in an MPTP model (23). Recent work identified a specific increase in the expression of glycoprotein non-metastatic B (GPNMB) in *GRN*-FTD patient brain lysate relative to both non-demented controls and other FTD related mutations (24). In addition, CSF from *GRN*-FTD patients showed increased amounts of GPNMB relative to non-demented controls (24). This indicates a specific increase in GPNMB expression with the loss of progranulin. When a mouse model of progranulin deficiency was investigated, an age-dependent increase in the expression of GPNMB was discovered. Brain lysate from young 3-month-old *Grn* knock-out (KO) mice did not show a significant change relative to age-matched wild-type (WT) controls, but 12-month-old *Grn* KO mice replicated the increase in GPNMB expression seen in the *GRN*-FTD patients (24). This gives further support that extended periods of insufficient progranulin results in increased GPNMB expression, likely in a compensatory mechanism(s) related to endo-lysosomal dysfunction.

Originally identified in a cancer cell line (25), GPNMB is a glycoprotein that is present both on the cell membrane and intracellularly in the autophagy pathway and is highly expressed on myeloid cells. ADAM/MMPs can cleave off the extracellular domain, generating a soluble extracellular fragment (ECF) (26), which has been demonstrated to serve a signaling role in a paracrine and an autocrine manner (27, 28). CD44 and Syndecan-4 are the published cell-surface receptors for GPNMB, but the effect of binding the GPNMB ECF is cell-type dependent (29, 30). Within the cell, GPNMB localizes with LC3+ vesicles, where it is believed to support the recruitment of the lysosome to autophagosomes (31), allowing for autophagic cargo to be successfully degraded. Given that progranulin has a profound effect on the endo-lysosomal pathway, it seems logically consistent that GPNMB would increase in an attempt to aid autophagolysosomal function. However, the mechanisms behind GPNMB regulation are not entirely well known. Microphthalmia-associated transcription factor (MITF) has been demonstrated to regulate GPNMB in dendritic cells (32), a class of immune cells with similar properties to macrophages.

Under basal conditions, mTORC1 phosphorylates the MiT/TFE transcription factor family of proteins (MITF, TFE3, TFEB, and TFEC), preventing their translocation to the nucleus by promoting interaction with the 14-3-3 cytosolic protein (33–35). In conditions with decreased mTORC1 activity, such as serum starvation and/or endo-lysosomal dysfunction, MiT/TFE transcription factors are not phosphorylated by mTORC1 and can drive the expression of CLEAR (Coordinated Lysosomal Expression and Regulation) genes (36), that push the cell towards increased autophagy and endo-lysosomal activity in an attempt to regain cellular homeostasis. However, there are a number of unknowns surrounding if and how these elements interact to contribute to the age-dependent increase in GPNMB expression in myeloid cells. This work seeks to close these gaps in knowledge by extending the investigation of GPNMB expression into the peripheral immune system and exploring candidate regulatory mechanism(s) that have been implicated to regulate GPNMB expression in the context of progranulin-deficiency.

## 2 Materials and Methods

### 2.1 Animals

C57BL/B6J mice and *Grn* KO (20) (The Jackson Laboratory, strain #:013175) mice were generated and maintained in the McKnight Brain Institute vivarium (University of Florida) at 22°C at 60-70% humidity with a 12-hour light/dark cycle and *ad libitum* access to standard rodent chow and water. C57BL/6J (B6) (Strain #000664) controls were used for all studies. All animal procedures were approved by the University of Florida Institutional Animal Care and Use Committee and were in accordance with the National Institute of Health Guide for the Care and Use of Laboratory Animals (NIH Publications No. 80-23, 8^th^ edition, revised 2011). Mice of both genotypes were co-housed and aged to 5-6 months.

On Day 1, WT and *Grn* KO mice were injected with 1mL of sterile 3% (m/v) BBL Thioglycollate Medium Brewer Modified (Becton Dickson, 211716) in diH2O. Mice also received Buprenorphine Sustained-Release (Zoopharm; Windsor, CO) every 48 hours for pain relief. On day 4, mice were sacrificed by cervical dislocation and their peritoneal membrane was exposed by carefully dissecting the skin and fur away from the midline. Great care was taken to avoid disrupting the integrity of the peritoneal membrane. 10mL of ice-cold RPMI media (Gibco #11875) was injected into the peritoneal cavity with a 27g syringe and gentle shaking was applied to loosen adherent cells into suspension. The media was then aspirated out with a 25g syringe and dispensed into a 15mL conical and placed on ice. After all of the cells were collected, the cell suspensions were filtered through a 70um pre-wet filter overlaid a 50mL conical and rinsed twice with ice-cold 1x HBSS -/- (Gibco #14175). The resulting suspension was spun at 400 x *g* for 5 minutes at 4°C. To remove red blood cells that may have contaminated the peritoneal cell suspension, the cell pellet was treated with 2mL of ACK Lysis Buffer (0.15 M NH_4_Cl 10mM KHCO_3_ 0.1mM EDTA) and gently vortexed until the pellet was resuspended. The ACK buffer was allowed to incubate until the red blood cells lysed, approximately 1-2 minutes. The reaction was quenched with 5mL of HBSS -/-, and the cell suspension was spun again at 400 x *g* for 5 minutes at 4°C. The resulting pellets were transferred into a sterile hood and the HBSS-ACK supernatant was aspirated away. The cells were resuspended in 3mL of warm complete RPMI media (Gibco RPMI #14175, 10% FBS, 1x Pen/Strep (Gibco #15140)) and counted and viability recorded using trypan-blue exclusion on an automated cell-counter (Countess™; Thermo). 2x10^6^ cells were plated in each well of a 6-well dish. Cells were incubated at 37°C, 5% CO_2_ for five to six hours to allow macrophages to adhere. Wells were washed twice with warm 1x DPBS -/- (Gibco #14190) to remove non-adherent peritoneal cells, leaving the adherent peritoneal macrophages behind. Warm complete RPMI media was applied to the macrophages until the experimental paradigm began the following day.

### 2.2 pMac treatment paradigms

ML329, an MITF inhibitor, (MedChemExpress, catalog number HY-101464) was reconstituted in DMSO under sterile conditions and diluted to a final concentration of 5uM and 10uM in warm RPMI media and applied for 24 or 48 hours. An equal volume of DMSO as the 10uM ML329 treatment was used as the vehicle control.

Recombinant mouse GPNMB ECD (Aviscera Biosciences, product code 00719-03-10) was diluted in pre-warmed complete RPMI to a final concentration of 0.5ug/ml and 1.0ug/mL and was applied for 4 hours. At the end of the treatment, the media that contained the GPNMB ECD was aspirated off of the cells. Pre-warmed 1x DBPS was used to wash the pMacs after the GPNMB-ECD treatment was finished to remove any remaining GPNMB-ECD. After the wash, lipopolysaccharide (LPS, from *Escherichia coli* O111:B4, Sigma-Aldrich) treatment was started. LPS-only and no-treatment controls were given a media change of fresh media at the same time. LPS was diluted to a final concentration of 1ug/mL, approximately 3,000 endotoxin units (EU), in pre-warmed complete RPMI media and applied to the macrophages for 3 hours. No treatment controls received an additional media change at the same time.

Recombinant mouse progranulin (R&D Systems, catalog #: 2557-PG) was diluted in complete RPMI media to a final concentration of 4 ug/mL and applied for 48 hours. No treatment controls received a media change at the same time.

### 2.3 Protein Fractionation and Western Blotting

At the end of the experimental paradigm(s), the plates containing the macrophages were removed from the incubator and placed on ice. Media was collected from select groups for downstream analysis before the macrophages were rinsed twice with ice-cold 1x PBS. After the PBS was aspirated off the second time, the cells were scraped up into the cytoplasmic fractionation buffer (150 mm sodium chloride, 50 mm Tris, pH 8.0, and 0.5% Triton X-100) with 1x protease and phosphatase inhibitors (c0mplete™ Protease Inhibitor Cocktail, Roche, product number 04693159001; PhosSTOP™, Roche, product number 04906837001). The resulting lysate was transferred to a labeled 1.5mL tube and placed on ice. Cell lysate was spun at 20,000 x *g* for 20 minutes at 4°C. After the spin was complete, the supernatant containing the soluble cytoplasmic proteins was transferred to a new labeled 1.5mL tube. The insoluble pellet was washed by adding the same volume of cytoplasmic fractionation buffer and vortexed briefly before being spun again at 13,000 x *g* for 20 minutes at 4°C. The supernatant was aspirated off, and the nuclear fractionation buffer (150 mm sodium chloride, 1.0% NP-40, 0.5% sodium deoxycholate, 0.1% SDS, and 50 mm Tris, pH 8.0) was added to the cell pellet. The pellet was briefly sonicated with a probe sonicator (QSonica, Q125 Sonicator, 125 Watts, 20 kHz) for 5 seconds and spun for a final time at 13,000 x *g* for 20 minutes at 4°C. The supernatant containing the soluble nuclear proteins was transferred to a separate labeled 1.5mL tube. The remaining pellet was discarded.

A BCA assay (Pierce™ BCA Protein Assay Kit, category number 23225) was used to determine the concentration of the resulting protein fractions. The indicated mass of protein was boiled for 5 minutes at 95°C with 4x Laemmeli Buffer (BioRad #1610747) supplemented with 2.5% (v/v) beta-mercaptoethanol and run on a 4-20% TGX gel (BioRad #5671094 and #5671095). Protein was transferred to polyvinylidene difluoride (PVDF) membrane (BioRad #1704273) using a Trans-Blot Turbo Transfer System (BioRad). Licor Revert 700 Total protein stain (Licor, P/N: 926-11011) was used to stain the total protein on the membrane and imaged on the Odyssey FC imaging system (Licor). Blots were incubated in primary antibodies (**Table 1**) overnight in 5% milk 1x TBS 0.1% Tween-20 pH 7.4. The blots were removed from primary and washed for five minutes in 1x TBS 0.1% Tween-20 pH 7.4. Blots were washed two more times for a total of three washes. The blots were incubated in secondary antibody in 5% Milk 1x TBS 0.1% Tween-20 pH 7.4 for 1 hour at RT with gentle rocking. After the 1-hour incubation, the blots were removed from the secondary antibody solution and washed as before three more times. To elicit signal from the HRP conjugated antibodies, SuperSignal West Femto Maximum Sensitivity Substrate (ThermoScientific™, category number 34096) reagents were combined at a 1:1 ratio. The reagents were quickly applied to the blot, and image collection began immediately. All images were collected using the Odyssey FC imaging system (Licor). Multiple images were collected for each blot with new Femto reagent applied after a short wash in 1x TBS 0.1% Tween-20 pH 7.4. ImageStudioLite was used to quantify the intensity of the bands. For the MITF and PGRN antibodies, fixation with 4% PFA in TBS for 10 minutes at RT was performed.

**Table 1:** List of primary and secondary antibodies for proteins of interest.

Target	Host/Source	Final Conc.	Manufacturer	Notes
Mouse GPNMB	Recombinant rabbit monoclonal antibody	1:2500	Abcam ab188222	
Mouse progranulin	Sheep Polyclonal	1:400	R&D Systems Af2557	Membrane fixed with 4% PFA-TBS prior to primary incubation
Mouse Histone H3	Rabbit polyclonal	1:1000	Cell Signaling Technology #9715	
Mouse GAPDH	Rabbit monoclonal antibody	1:2500-5000	Cell Signaling Technology 14C10	1:2500 dilution was applied to the membranes after it was confirmed that the MITF signal was successfully stripped off. 1:5000 applied a
Mouse MITF	Rabbit monoclonal antibody	1:1000	Cell Signaling Technology D3B4T	Membrane fixed with 4% PFA-TBS prior to primary incubation
Goat anti-Rabbit poly HRP	Goat antibody	1:20,000	Jackson Immuno Research 111-035-003	
Donkey anti-Sheep poly HRP	Donkey antibody	1:20,000	Invitrogen A16041	

The membrane was stripped between the probe of MITF and GAPDH using a buffer containing a final concentration of 1 M Tris, 20% SDS, and 0.7% BME. The solution was pre-warmed to 50°C and applied to the membrane for 15 minutes at 50°C. The membrane was blocked in 5% Milk TBS- Tw for 30 minutes after stripping. An additional probe with Femto reagent was performed to determine the success of the stripping protocol. Signal was normalized to the surrounding background before being normalized to GAPDH or H3 loading control for each band.

### 2.4 RNA and qPCR

RNA was isolated using a Qiagen Mini-kit (Qiagen, category number 74104), following manufacturer instructions with minimal alterations. The concentration of BME was increased to a final concentration of 20uM to inhibit endogenous RNase activity. After elution, RNA concentration was determined using a Denovix spectrophotometer. cDNA was generated using a High-Capacity cDNA Reverse Transcription Kit (Applied Biosystems™, catalog number 4374966) following kit instructions.

2x Universal SYBR Green Fast qPCR Mix (Abclonal, RK21203) was used with the indicated primers (**Table 2**) for detection.

**Table 2:** List of qPCR primers for genes of interest.

Gene	Forward	Reverse
*Il-1b*	CAA CCA ACA AGT GAT ATT CTC CAT G	GAT CCA CAC TCT CCA GCT GCA
*Il-6*	GAG GAT ACC ACT CCC AAC AGA CC	AAG TGC ATC ATC GTT GTT CAT ACA
*Il-10*	GGT TGC CAA GCC TTA TCG GA	ACC TGC TCC ACT GCC TTG CT
*Gpnmb*	AAG CGA TTT CDG GAT GTG CT	CTT CCC AGG AGT CCT TCC AC
*Tnf*	CTG AGG TCA ATC TGC CCA AGT AC	CTT CAC AGA GCA ATG ACT CCA AAG
*Ctsd* (Origene, NM_009983)	TAA GAC CAC GGA GCC AGT GTC A	CCA CAG GTT AGA GGA GCC AGT A
*Gapdh*	CAA GGT CAT CCA TGA CAA CTT TG	GGC CAT CCA CAG TCT TCT GG

All primer sequences were generated and validated in house, except the *Ctsd* primer set, which was sourced from Origene (NM_009983). To generate primers, the University of Santa Cruz Genomics Institute Genome Browser was used to identify exon spanning sequences for the genes of interest in the appropriate species. Multiple exon spanning sequences were selected and the NIH BLAST tool was used to check for specificity. Next, the spanning sequences were copied into the online Primer3Plus tool. Candidate primer sequences were generated and reviewed for the following criteria: primer length between 20-27 bases, product length is around 100bp, GC content is 40-60%, and the Tm difference between the primers is less than 4°C. The reviewed sequences were ordered as custom oligos from IDT with standard desalting purification.

Once the primer sequences arrived, the tubes were briefly spun down to collect the powder in the bottom of the tube. The indicated amount of nuclease-free ultrapure water was added to each tube to generate 100µM stock. The primers were vortexed for 15 seconds to reconstitute them. Working stock concentrations were generated by adding 12.5uL of each forward and reverse primer for each gene into 975uL of nuclease-free ultrapure water. The working stocks were made homogenous with a brief vortex.

To validate in-house primer sequences: a five-fold dilution series was generated using cDNA from a source with high expression of the genes of interest. qPCR was plated to determine how Ct values change with decreased cDNA concentration. A cyclophilin primer set was used as a positive control for amplification and the standard curve was generated by plotting the fold change of the mass of cDNA against the detected Ct values for each primer set. A primer is considered valid if the R value is 0.97 or higher and the slope of the line is m=-3.3 ± 0.1.

Plate qPCR to determine how the Ct value changes with decreasing cDNA concentration. Use a cyclophilin primer set as a positive control for amplification and the standard curve generated by plotting the fold change in the mass of cDNA and the detected Ct value (example graph below). A primer is considered valid if the R^2^ value is 0.97 or higher and the slope of the line is m=-3.3 ± 0.1.

A final mass of 6.25ng of cDNA and a final concentration of 0.15µM of each forward and reverse primer was used per well. The PCR data was collecting using a Quantstudio5 system with the following cycle: 50°C for 2 minutes, 95°C for 10 minutes, 40 cycles (95°C for 15 seconds and 60°C for 1 minute), 95°C for 15 seconds, 60°C for 15 seconds, and 95°C for 55 seconds. Each sample was run in triplicate per gene. Samples with variance beyond 0.4 standard deviations from the sample’s average were excluded.

### 2.5 ELISA

Conditioned media was collected from the pMacs at the indicated times. After being transferred to a 1.5mL tube, the media was centrifugated at 10,000 x *g* for 10 minutes at 4°C to remove any cell debris. The supernatant was transferred to a new tube and stored at -20°C for later analysis. Endogenous GPNMB ECF was measured using a Biotechne GPNMB ELISA Kit (Biotechne/R&D Systems, DY2330) following manufacturer instructions with minor modifications. A four-parameter logistic(4-PL) curve was generated from the GPNMB ECF standards included in the kit. The standard curve was created with an online 4-PL curve calculator from AAT Bioquest (https://www.aatbio.com/tools/four-parameter-logistic-4pl-curve-regression-online-calculator). The equation from the 4-PL curve was used to determine the concentration of GPNMB ECF in the test samples.

## 3 Results

### 3.1 GPNMB is dysregulated in *Grn* KO pMacs

**Figure 1:**
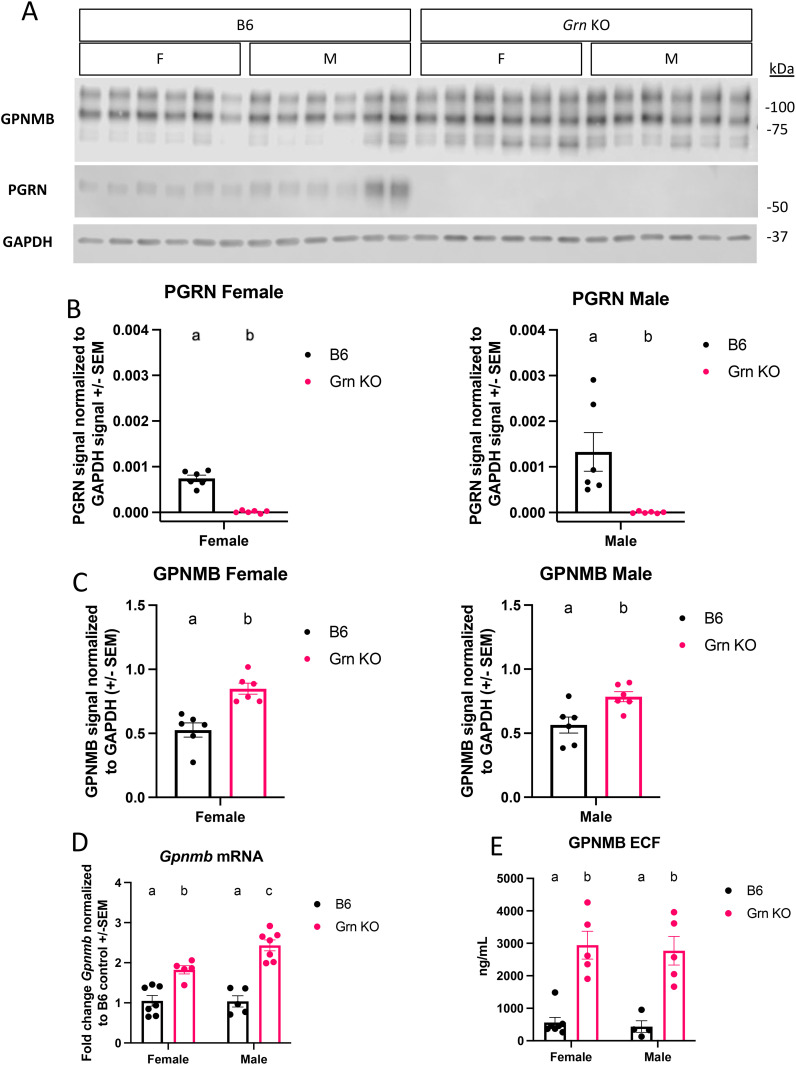
GPNMB protein is overproduced in *Grn* KO pMacs **(A)** Representative western blot images of GPNMB, PGRN, and GAPDH from B6 and *Grn* KO pMacs **(B)** Quantification of PGRN signal normalized to GAPDH. **(C)** Quantification of GPNMB signal normalized to GAPDH, n=6 per sex and genotype, n = 5-6 per sex and genotype, unpaired t-test, bars represent mean +/- SEM. **(D)** qPCR data on baseline *Gpnmb* transcript from bothB6 and *Grn* KO pMac, n=5-7 per sex and genotype, two-way ANOVA with Sidak’s multiple comparisons test. **(E)** GPNMB ELISA measurement of GPNMB ECF in culture media generated by pMacs over 48 hours, n=4-5 per sex and genotype, two-way ANOVA with Sidak’s multiple comparisons test. Letter(s) above the bar graphs represent the results of the *post-hoc* tests. Groups that share the same letter are not significantly different from one another.

Previous work identified a novel increase in GPNMB signal in brain-resident cells, presumed to be microglia, in *Grn* KO mice beginning at 12-months old (24). An age series allowed the authors to conclude that there is an age-dependent increase in GPNMB signal in the central nervous system (CNS) of *Grn* KO mice. To extend on this finding, we investigated the relative abundance of GPNMB in peripheral myeloid cells by isolating thioglycollate-elicited peritoneal macrophages (pMacs) from *Grn* KO mice. Western blot analysis confirmed the absence of progranulin and also showed a significant increase in GPNMB protein in pMacs collected from 5–6-month-old *Grn* KO mice relative to B6 controls in both male and female mice (**Figures 1A–C**). All three bands present in the probe for GPNMB were quantified. The lowest band, approximately 68 kDa, is the nascent protein, and the two higher weight bands, around 95 and 110 kDa, have been glycosylated and may have additional post-translational modifications.

However, it was previously unclear if the relative increase in GPNMB protein abundance was a non-specific intracellular accumulation as a result of endo-lysosomal dysfunction without sufficient progranulin expression. Using qPCR, we determined that pMacs from 5-6-month-old *Grn* KO mice had a significant increase in *Gpnmb* mRNA relative to B6 controls, with male *Grn* KO pMacs expressing significantly higher amounts than female *Grn* KO pMacs. (**Figure 1D**). This supports that this increase in GPNMB protein is a result of increased expression and not an endo-lysosomal bystander. Previous work has established that GPNMB is subject to cleavage events by extracellular proteases like ADAM10 that produces a soluble extracellular fragment (ECF) (26). Given the increase in GPNMB protein substrate, we investigated the amount of GPNMB ECF that was produced in pMac media. Using an ELISA specific for endogenous GPNMB ECF, we determined that there is a significant increase in the amount of GPNMB ECF generated in pMacs collected from 5–6-month-old male and female *Grn* KO mice relative to B6 controls (**Figure 1E**). Together, this data demonstrates that there is an active increase in both GPNMB protein and the soluble ECF generated from it, in progranulin-deficient macrophages.

### 3.2 *Grn* KO pMacs show diminished expression of early inflammatory genes

Having discovered an increase in the GPNMB ECF generated by *Grn* KO pMacs, we wanted to investigate any functional effect of recombinant GPNMB extracellular domain (ECD) treatment on the behaviour of these pMacs. Previous work has identified a decrease in inflammatory gene expression with recombinant GPNMB ECD treatment in *Gpnmb* KO pMacs (28). We adapted their paradigm to our own purposes, pre-treating two groups of cells with two doses of recombinant GPNMB ECD for four hours prior to three hours of LPS stimulation with an LPS only and no treatment control. This paradigm allows us to determine the differences in inflammatory gene expression between *Grn* KO and B6 pMacs during LPS stimulation, but it also permits us to detect any changes in inflammatory gene expression with GPNMB ECD pre-treatment.

**Figure 2:**
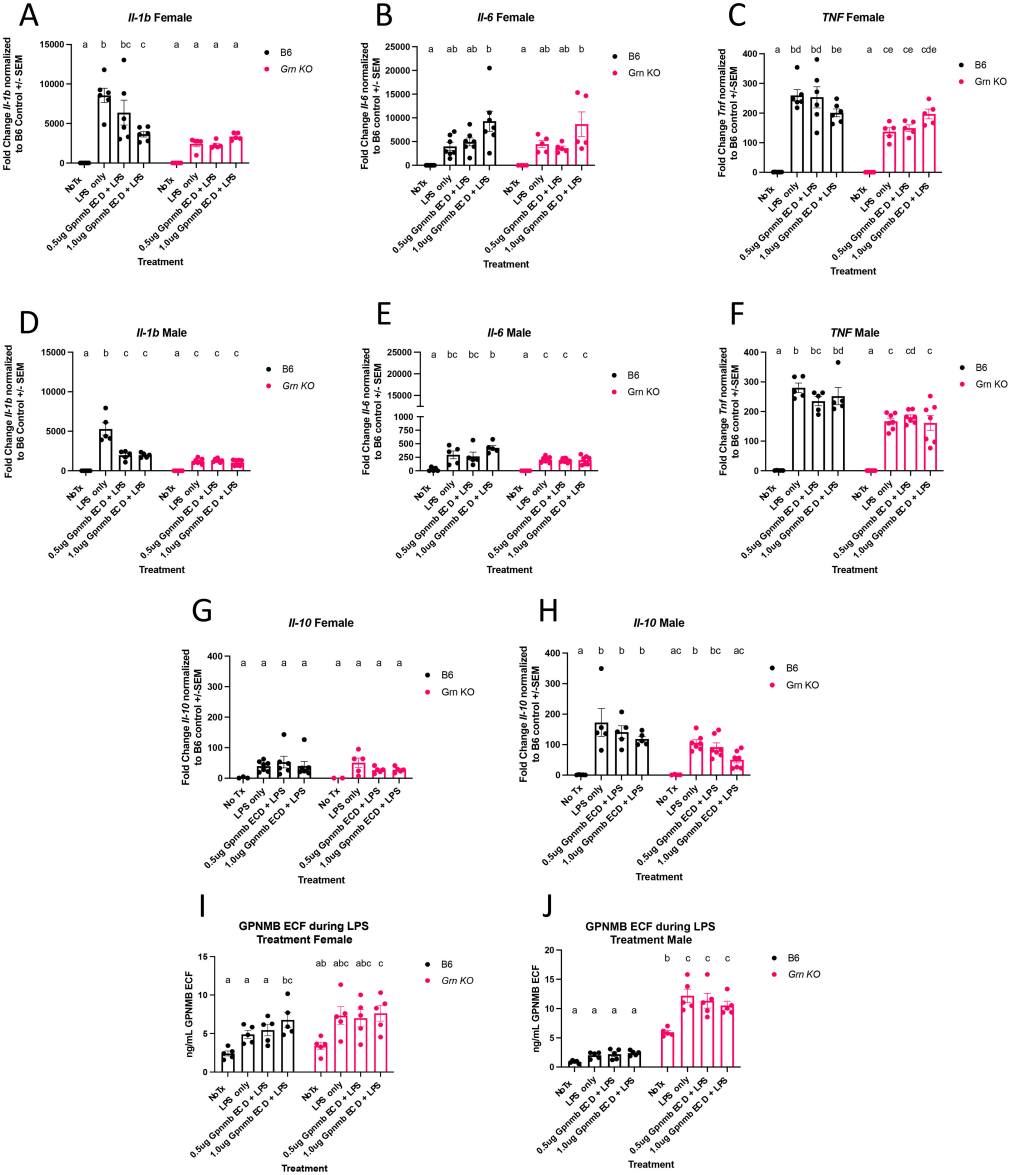
*Grn* KO pMacs display reduced LPS-dependent expression of early inflammatory genes **(A)** qPCR for *Il-1b* expression in female B6 and *Grn* KO pMacs. **(B)** qPCR for *Il-6* expression in female B6 and *Grn* KO pMacs. **(C)** qPCR for *Tnf* expression in female B6 and *Grn* KO pMacs. **(D)** qPCR for *Il-1b* expression in male B6 and *Grn* KO pMacs. **(E)** qPCR for *Il-6* expression in male B6 and *Grn* KO pMacs. **(F)** qPCR for *Tnf* expression in male B6 and *Grn* KO pMacs. **(G)** qPCR for *Il-10* expression in female B6 and *Grn* KO pMacs. **(H)** qPCR for Il-10 expression in male B6 and *Grn* KO pMacs. **(I, J)** GPNMB ELISA measurement of GPNMB ECF in culture media generated by pMacs during experimental paradigm. Two-way ANOVA with Sidak’s multiple comparisons test for every panel, n = 5-7, bars mean represent +/- SEM. Groups that share the same letter are not significantly different from one another.

qPCR analysis demonstrated multiple notable differences in the expression of inflammatory genes between genotypes. *Il-1b* transcript was increased with LPS only stimulation in both sexes of B6 pMacs, but there was a diminished though significant increase in *Il-1b* transcript in *Grn* KO male pMacs and no change at all in *Grn* KO female pMacs with LPS only stimulation (**Figures 2A, D**). There was no significant increase in *Il-6* transcript abundance in B6 and *Grn* KO female pMacs stimulated with only LPS relative to no treatment controls, but males of both genotypes had a significant, if smaller, increase in *Il-6* transcript with only LPS stimulation (**Figures 2B, E**). *Tnf* transcript was also significantly different between the genotypes following only LPS stimulation, but neither sex significantly differed from their respective B6 controls (**Figures 2C, F**). In contrast, no differences in *Il-10* transcript between any genotype of either sex were observed (**Figures 2G, H**).

The effect of the GPNMB ECD pre-treatment is less impactful than the genotype. In both male and female B6 pMacs, there is significantly decreased *Il-1b* transcript relative to the LPS only control, suggesting that GPNMB ECD pre-treatment was sufficient to blunt the LPS-mediated increase (**Figures 2A, D**). Additionally, female pMacs from both genotypes show no significant increase in *Il-6* transcript with LPS stimulation relative to the no treatment control except for when pre-treated with 1ug/mL of GPNMB ECD (**Figure 2B**). However, no significant effect of GPNMB pre-treatment on *Il-6* was observed in pMacs from B6 males (**Figure 2E**). *Tnf* transcript levels did not significantly change with pre-exposure to either dose of GPNMB ECD (**Figures 2C, F**). Finally, *Il-10* transcript showed no change due to GPNMB ECD treatment, with the exception of the *Grn* KO males, where both doses of GPNMB ECD reduced the *Il-10* transcript to levels that are not significantly different from the no treatment control (**Figure 2H**).

Finally, we investigated if GPNMB ECD pre-treatment and/or LPS only treatment altered the amount of GPNMB ECF that was released into the media by pMacs. In both genotypes of female pMacs, GPNMB ECF concentration increased with LPS but did not reach statistical significance until pre-treated with 1ug of GPNMB ECD (**Figure 2I**). In contrast, the B6 male pMacs did not change the amount of GPNMB ECF with any treatment, but the *Grn* KO pMacs generated a significant increase in GPNMB ECF with LPS stimulation regardless of GPNMB ECD pre-treatment (**Figure 2J**). Collectively, this data demonstrates a key significant decrease in critical pro-inflammatory cytokine expression in *Grn* KO pMacs, and the ability of GPNMB ECD to blunt the LPS-mediated increase in *Il-1b* expression in B6 pMacs.

### 3.3 Re-addition of progranulin rescues GPNMB phenotype in *Grn* KO pMacs

**Figure 3:**
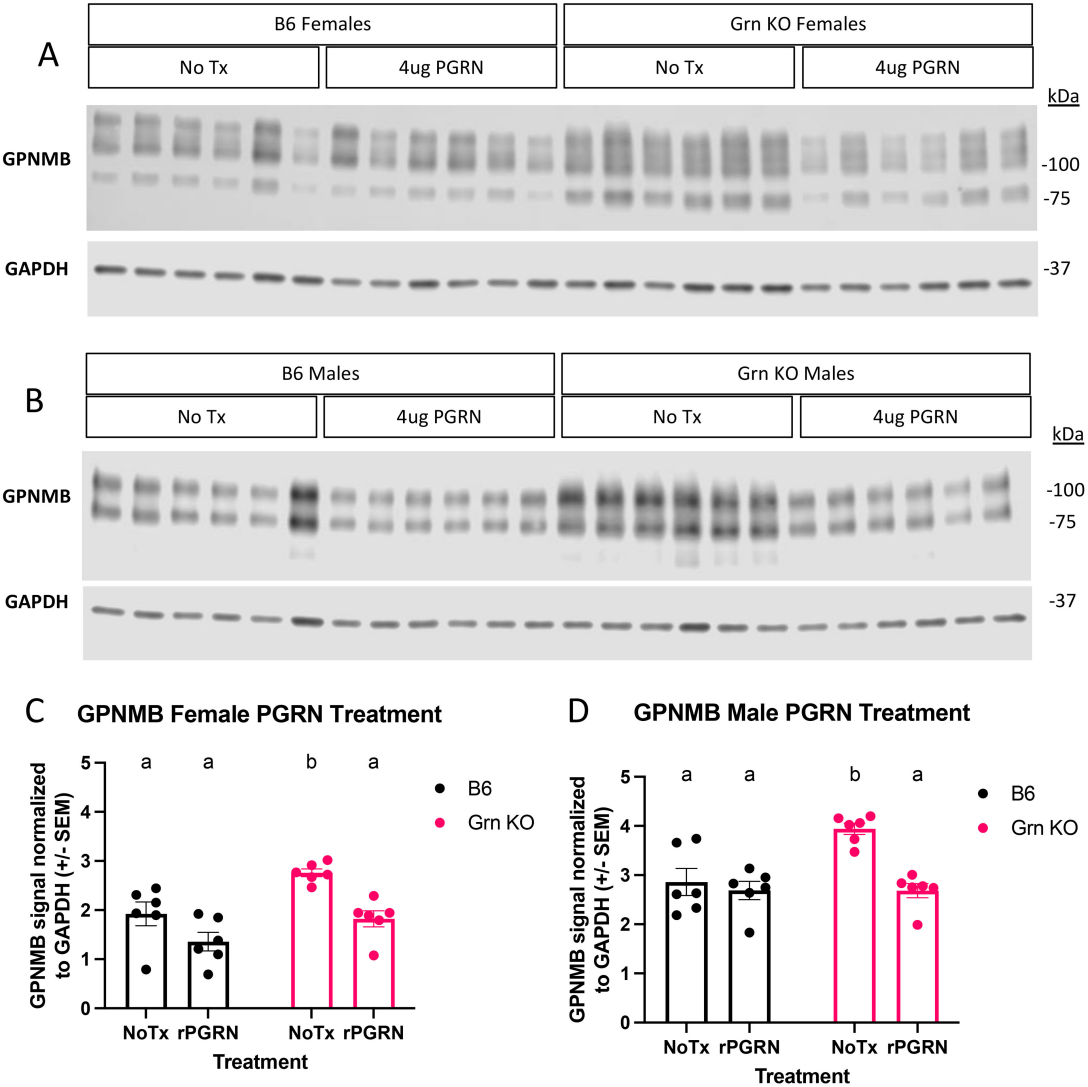
Re-addition of progranulin protein rescues GPNMB dysregulation in *Grn* KO pMacs **(A)** Representative western blot images of GPNMB and GAPDH from female B6 and *Grn* KO pMacs with and without 48 hours of 4ug/mL of recombinant progranulin treatment. **(B)** Representative western blot images of GPNMB and GAPDH from male B6 and *Grn* KO pMacs with and without 48 hours of 4ug/mL of recombinant progranulin treatment. **(C)** Quantification of GPNMB signal normalized to GAPDH. **(D)** Quantification of GPNMB signal normalized to GAPDH. Two-way ANOVA with Sidak’s multiple comparisons test, n = 6 per genotype, bars represent mean +/- SEM. Groups that share the same letter are not significantly different from one another.

To investigate the regulation of GPNMB in progranulin-deficient macrophages, we treated both B6 and *Grn* KO pMacs with recombinant mouse progranulin. Using western blot analysis, we determined that the relative abundance of GPNMB protein was significantly increased in Grn KO pMacs that did not receive progranulin but was not significantly different from B6 controls after treatment with recombinant progranulin (**Figures 3A–D**). We detected no significant change in GPNMB with the addition of exogenous progranulin in B6 pMacs (**Figures 3A–D**). This data suggests that the re-addition of progranulin is sufficient to significantly decrease GPNMB levels in *Grn* KO pMacs, but the exogenous progranulin had no significant effects on the expression of GPNMB in B6 pMacs.

### 3.4 MITF is dysregulated in *Grn* KO pMacs

**Figure 4:**
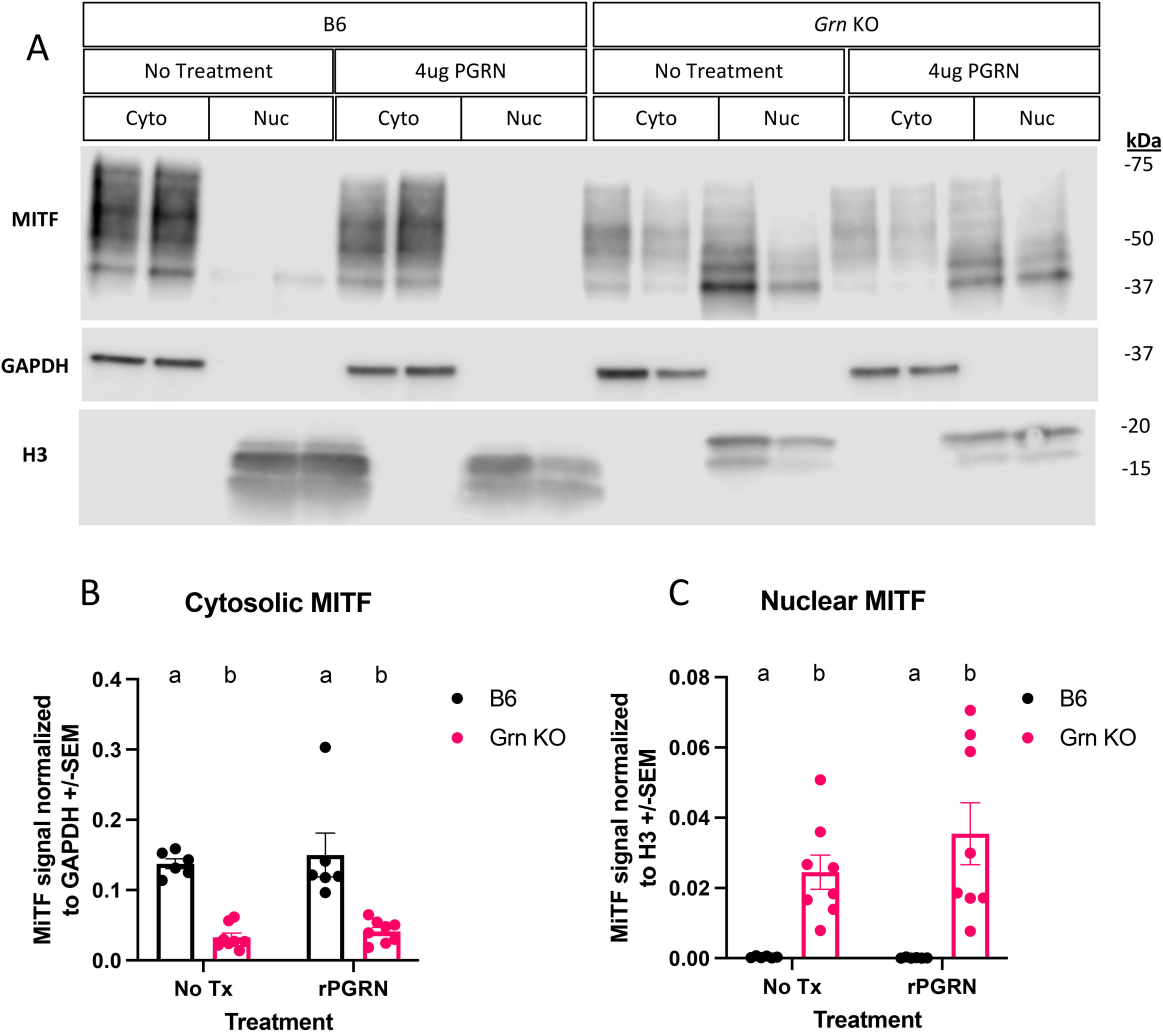
MITF localization is primarily nuclear in *Grn* KO pMacs and unresponsive to progranulin re-addition. **(A)** Representative western blot images of MITF, GAPDH, and H3 from B6 and *Grn* KO pMacs with and without 48 hours of 4ug/mL of recombinant progranulin treatment. **(B)** Quantification of MITF signal normalized to GAPDH signal. **(C)** Quantification of MITF signal normalized to H3 signal. Two-way ANOVA with Sidak’s multiple comparisons test, n = 6 per genotype, bars represent mean +/- SEM. Groups that share the same letter are not significantly different from one another.

Previous reports have implicated the transcription factor MITF to regulate GPNMB in dendritic cells (32), RAW264.7 cells (37), and osteoclasts (37). Given our observation of increased GPNMB expression in *Grn* KO pMacs, we investigated the regulation of MITF in both the presence and absence of recombinant progranulin treatment (**Figure 3**). To best determine the relative abundance of MITF, we used biochemical fractionation to concentrate nuclear and cytosolic proteins into separate lysates. Subsequent western blot analysis identified distinct patterns of MITF abundance in the nuclear and cytosolic fractions of B6 and *Grn* KO pMacs. B6 pMacs have a greater amount of MITF in the cytosolic fraction but very little in the nuclear fraction (**Figures 4A–C**). In contrast, *Grn* KO pMacs have little cytosolic MITF relative to B6, but instead have greater nuclear MITF relative to B6 pMacs. Re-addition of recombinant progranulin for 48 hours did not rescue the dysregulated nuclear MITF in *Grn* KO pMacs (**Figures 4A, C**).

**Figure 5:**
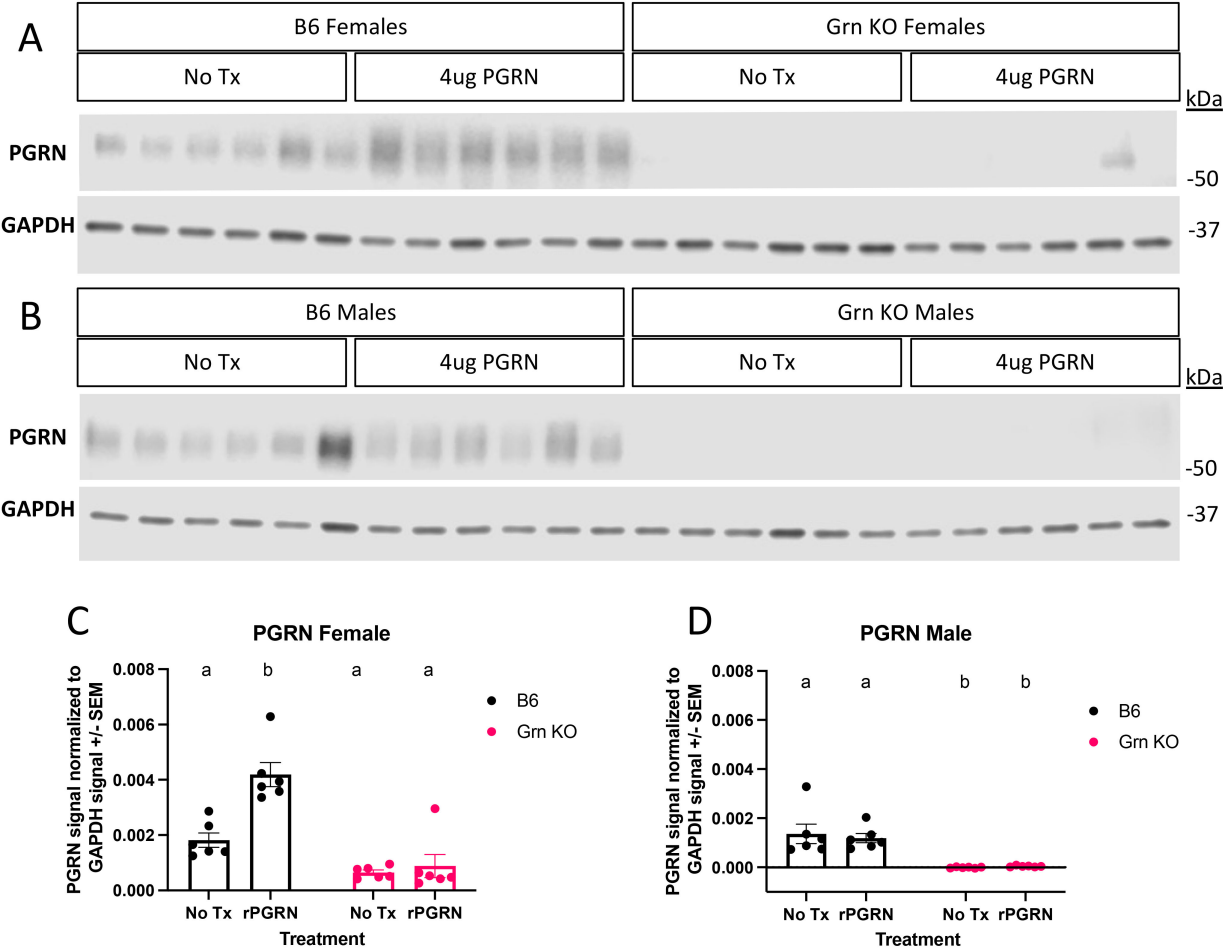
Recombinant progranulin is processed with faster kinetics in *Grn* KO pMacs relative to WT pMacs. **(A)** Representative western blot images of PGRN and GAPDH signal from pMacs collected from female B6 and *Grn* KO with and without 48 hours of 4ug/mL of recombinant progranulin treatment. **(B)** Representative western blot images of PGRN and GAPDH signal from pMacs collected from male B6 and *Grn* KO with and without 48 hours of 4ug/mL of recombinant progranulin treatment. **(C)** Quantification of PGRN signal normalized to GAPDH signal from female B6 and *Grn* KO pMacs with and without 48 hours of 4ug/mL recombinant progranulin treatment. **(D)** Quantification of PGRN signal normalized to GAPDH signal from male B6 and *Grn* KO pMacs with and without 48 hours of 4ug/mL recombinant progranulin treatment. Two-way ANOVA with Sidak’s multiple comparison test, n=6 per genotype, per treatment, bars represent mean +/-SEM. Groups that share the same letter are not significantly different from one another.

This lack of MITF rescue was initially surprising, but to better understand the underlying dynamics, we performed western blot analysis for progranulin itself on the cohort of pMacs treated with recombinant progranulin (**Figure 5**). In the 48 hours following the re-addition of progranulin, both sexes of *Grn* KO pMacs have almost completely degraded the input progranulin and show little signal beyond background in the no treatment group (**Figures 5A–D**). In contrast, the female B6 pMacs that were treated with exogenous progranulin have significantly elevated cytosolic progranulin levels (**Figures 5A, C**). B6 male pMacs given exogenous progranulin had no significant change from the no treatment baseline (**Figures 5B, D**). The lack of MITF rescue in the *Grn* KO pMacs (**Figure 3**, **Figure 4**) correlates with the lack of available intact progranulin (**Figure 5**). While GPNMB was rescued with 48 hours of recombinant progranulin treatment (**Figure 3**), the timepoint and/or dose of progranulin was insufficient to demonstrate a progranulin-mediated rescue in MITF localization in *Grn* KO pMacs (**Figures 4**, **5**).

### 3.5 MITF-inhibition does not rescue the GPNMB phenotype in *Grn* KO pMacs

**Figure 6:**
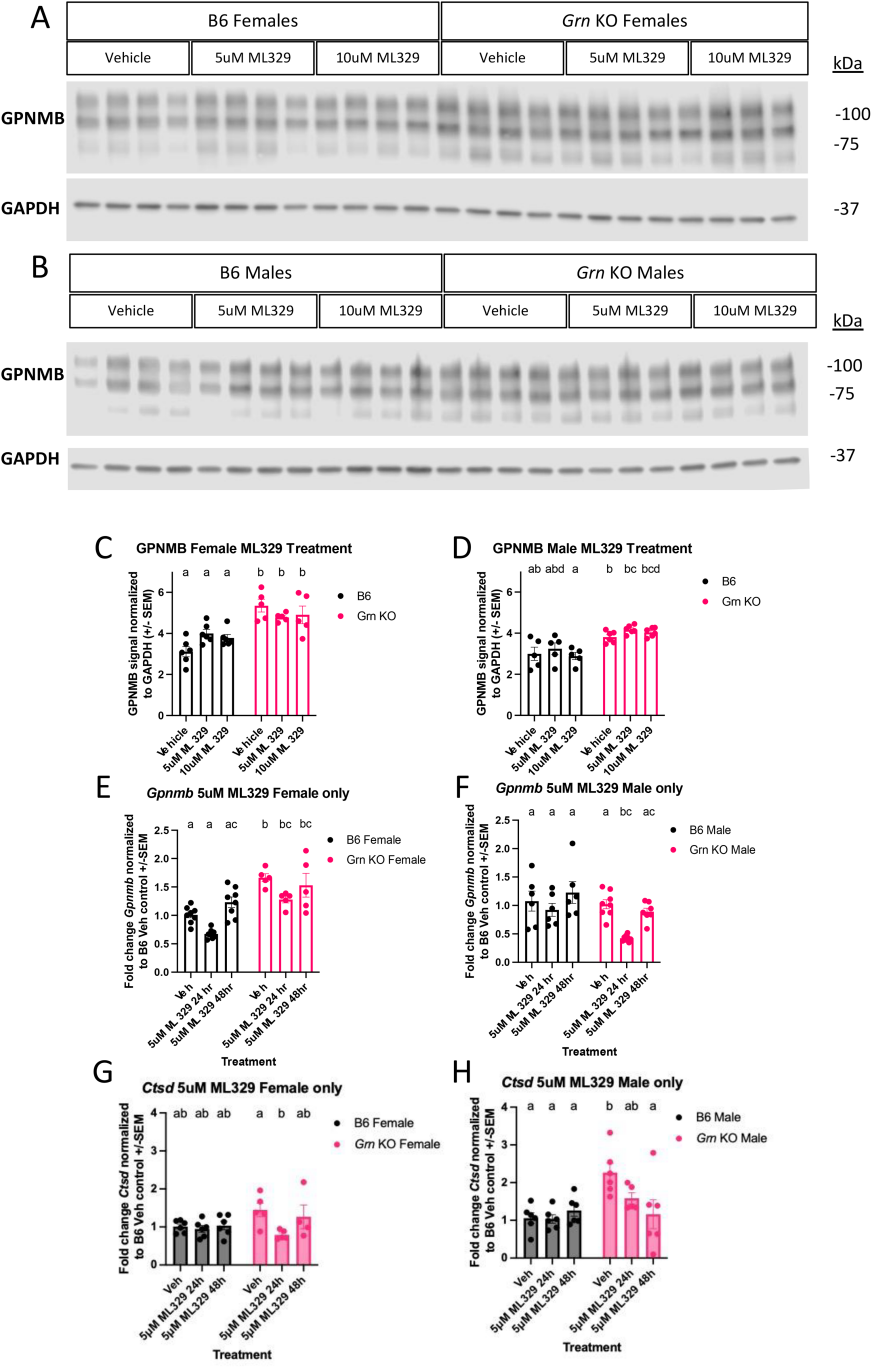
Inhibition of MITF activity alone does not rescue the overproduction of GPNMB transcript or protein in *Grn* KO pMacs **(A)** Representative western blot images of B6 and *Grn* KO female pMacs treated with vehicle (DMSO), 5uM, or 10uM of ML329 for 24 hours. **(B)** Representative western blot images of B6 and *Grn* KO male pMacs treated with vehicle (DMSO), 5uM, or 10uM of ML329 for 24 hours. **(C)** Quantification of GPNMB signal normalized to GAPDH from female pMacs, n=-5-6 per genotype and treatment. Two-way ANOVA with Sidak’s multiple comparisons test. **(D)** Quantification of GPNMB signal normalized to GAPDH from male pMacs, n=-5-6 per genotype and treatment, bars represent mean +/- SEM. Two-way ANOVA with Sidak’s multiple comparisons test. **(E)** qPCR for *Gpnmb* expression in female B6 and *Grn* KO pMacs, n=5-8 per genotype per treatment. **(F)** qPCR for *Gpnmb* expression in male B6 and *Grn* KO pMacs, n=5-8 per genotype per treatment, bars represent mean +/-SEM. Two-way ANOVA with Sidak’s multiple comparisons test. **(G)** qPCR for Ctsd expression in female B6 and *Grn* KO pMacs, n=5-8 per genotype per treatment, bars represent mean +/-SEM. **(H)** qPCR for Ctsd expression in male B6 and *Grn* KO pMacs, n=5-8 per genotype per treatment, bars represent mean +/-SEM. Two-way ANOVA with Sidak’s multiple comparisons test. Groups that share the same letter are not significantly different from one another.

The lack of significant effect of recombinant progranulin on MITF localization in pMacs prompted us to question the involvement of MITF in the regulation of GPNMB in this model. To investigate this further, we treated pMacs with an MITF inhibitor, ML329, for 24 hours prior to lysate collection. Western blot analysis indicated that, relative to DMSO vehicle control, neither dose of ML329 significantly changed the abundance of GPNMB in any genotype or sex within 24 hours of addition (**Figures 6A–D**). However, to rule out the possibility that the half-life of GPNMB in an unstimulated state was longer than 24 hours, we repeated the experiment by treating with 5uM of ML329 for either 24 or 48 hours, collecting RNA and probing for changes in *Gpnmb* and, a CLEAR gene control, *Ctsd* mRNA via qPCR. At 24 hours of 5uM ML329 treatment, *Gpnmb* transcript levels were appreciably decreased but did not reach the threshold of statistical significance relative to DMSO vehicle control, with the exception of *Grn* KO males (**Figures 6E, F**). However, at the 48-hour timepoint, *Gpnmb* transcript levels were comparable to vehicle control levels in all groups (**Figures 6E, F**). For *Cstd* transcription, ML329 had no significant effect on B6 pMacs, but did have a significant decrease in *Ctsd* transcription in *Grn* KO pMacs with 24 hours of treatment (**Figures 6G, H**). At 48 hours of treatment, *Grn* KO pMacs isolated from female mice had levels of *Ctsd* expression insignificantly different from vehicle controls, but *Grn* KO pMacs isolated from males showed further decrease *Ctsd* transcripts. Together, these data suggest MITF activity may contribute to the GPNMB phenotype in *Grn* KO pMacs but is ultimately not required for the increased GPNMB expression in the absence of progranulin, suggesting redundancy in the regulatory cascade of GPNMB production and other CLEAR genes.

## 4 Discussion

Overall, our work demonstrates, for the first time, that GPNMB is dysregulated in peripheral macrophages in the absence of progranulin at a significantly earlier time point than previously reported for its dysregulation in the CNS in proganulin-deficient mice. Furthermore, our findings reveal that this increase in GPNMB is driven by increased expression of *Gpnmb* transcript and dynamically regulated by progranulin levels. Finally, we report another novel finding involving MITF dysregulation in *Grn* KO pMacs that correlates with, but does not appear to be required for, the dysregulated expression of GPNMB in progranulin-deficiency. Taken together, these data demonstrate the vital role of progranulin in modulating the peripheral immune environment and also raise the interesting possibility that events in the periphery may pre-empt those in the CNS in progranulin-deficient states. These findings provide new insight into the mechanisms involved in dysregulation of central-peripheral neuroimmune crosstalk and may represent unique opportunities for early intervention by targeting the periphery to delay or prevent dysregulation in the CNS.

First, the studies presented herein not only confirm the phenotype of increased GPNMB expression in progranulin-deficient myeloid cells reported previously (24) but also extend these observations into the peripheral immune system, revealing increases in GPNMB at a significantly earlier time point than previously reported for microglia in the CNS. Additionally, our data demonstrate that this increase is a dynamic and possibly compensatory response to insufficient progranulin levels that is able to be resolved with restoration of sufficient progranulin treatment and also results in a significant increase in the concentration of the soluble signaling fragment generated from GPNMB (GPNMB ECF) which has been reported to have anti-inflammatory effects (27, 28). Therefore, to investigate the potential effects of elevated GPNMB ECF on macrophage behaviour in response to the loss of progranulin expression, we adapted a paradigm involving pre-treatment with a recombinant fragment of the extracellular domain of GPNMB (GPNMB ECD) prior to LPS stimulation (28). First, when comparing the effects of LPS stimulation to LPS-untreated controls, we found that *Grn* KO pMacs overall contained reduced transcripts for classic inflammatory genes relative to B6 pMacs. This may seem counterintuitive, as progranulin is typically associated with anti-inflammatory effects, and previously published results suggest increased expression of inflammatory genes in response to LPS-stimulation, but caution is advised with interpreting an early snapshot of the inflammatory cascade as an accurate representation of the integrated immune response. Specifically, robust inflammatory responses are necessary to maintain organismal health but requires strict regulation to prevent deleterious outcomes. In our view, this lagging response to LPS stimulation in the *Grn* KO pMacs is an indication of the dysfunction related to insufficient progranulin levels.

It’s possible that the lack of sufficient progranulin resulted in altered inflammatory dynamics, where the peak of inflammation is at a later point in LPS stimulation, and *Grn* KO pMacs do generate more inflammatory markers than B6. Alternatively, it’s possible that insufficient progranulin levels prevents or modifies the regulation of inflammation, and while *Grn* KO pMacs do not have a higher expression of inflammatory genes than B6 pMacs at any specific point, the aggregate expression of inflammatory genes is greater in the *Grn* KO relative to the B6. An additional factor to consider is the method of generating pMacs for study. Macrophage biologists typically utilize bone marrow derived macrophages (BMDMs) or pMacs for *ex vivo* assessment of the innate immune system. We used thioglycolate-elicited pMacs for our work to increase macrophage yield in the peritoneum which can then be harvested without the need for further differentiation *ex vivo*, an advantage over the use of BMDMs, whereby bone marrow is isolated from mouse femurs, and stem cells are further differentiated in culture to macrophages with major colony stimulation factor (M-CSF) or conditioned media from the mouse fibroblast cell line L929 (20, 38). pMacs therefore provide a readout of the responsiveness of the innate immune system in the background of the animal from which they are isolated, of particular importance for knockout or genetically modified animals when assessing effects of genotype at different ages (39). Differences between our results and other published results are likely the result of different methods of macrophage isolation and culturing as well as differences in the dose and duration of LPS stimulation.

Next, pre-treatment of B6 pMacs with GPNMB ECD prior to LPS stimulation achieved a blunting of select pro-inflammatory genes like *Il-1b* in both sexes. Other early inflammatory genes did not appear to be significantly affected by the GPNMB ECD pre-treatment in B6 pMacs, but it is known that peak expression for inflammatory genes following LPS treatment or an immune challenge differ significantly (40, 41), so a single time point is insufficient to make a definitive conclusion about the possible effects that GPNMB ECD pre-treatment may have on the kinetics of inflammatory gene expression. In contrast, there was no significant effect of GPNMB ECD pre-treatment in *Grn* KO pMacs with or without LPS challenge. However, we found that *Grn* KO pMacs generate significantly more GPNMB ECF than B6 pMacs while at rest; therefore, it is possible that the GPNMB ECF generated into the culture media between the time the cells were plated and the beginning of the stimulation paradigm affected this response and should be taken into account in the interpretation of the effects of exogenously added GPNMB ECF on inflammatory genes generated following LPS stimulation. It is possible that this inevitable “pre-pre-treatment” desensitized the *Grn* KO pMacs to any possible further response to additional GPNMB ECD administered during the LPS stimulation paradigm. Additionally, this effect may be responsible for the diminished inflammatory responses noted in *Grn* KO pMacs. Future studies will focus on addressing these and other possibilities.

Importantly, we successfully demonstrate that progranulin re-addition is sufficient to rescue the increase in GPNMB protein in *Grn* KO pMacs, but we ruled out that normalization of MITF activity is required for this rescue. Specifically, based on previous work highlighting the role of MITF in driving the expression of CLEAR genes and the ability of MITF to drive GPNMB expression in dendritic cells, we sought to test the hypothesis that MITF was involved in the rescue mechanism. In support of our hypothesis, we found clear differences in the basal regulation of MITF in B6 versus *Grn* KO pMacs. Specifically, western blots of cytosolic and nuclear fractions revealed a clear and significant decrease in cytosolic MITF and a corresponding increase in nuclear MITF in *Grn* KO pMacs relative to B6 controls. We interpret this to represent altered MITF regulation in *Grn* KO pMacs. To further interrogate the involvement of MITF in the progranulin-mediated rescue of GPNMB expression, we then compared the baseline fractions to fractions taken from B6 and *Grn* KO pMacs treated with recombinant mouse progranulin. The same treatment was able to rescue the GPNMB phenotype in *Grn* KO pMacs, but there was no significant shift in the ratio of nuclear or cytosolic MITF in *Grn* KO pMacs that received the same treatment, suggesting that although MITF may be involved in the rescue, translocation of MITF is not required to mediate the progranulin-mediated reduction in GPNMB expression. Therefore, the precise mechanism behind this rescue remains unclear, but there may be several reasons for this outcome. First, it is possible that 48 hours of 4ug/mL of progranulin treatment was not a sufficient length of time or dose of progranulin to allow significant clearance of MITF from the nucleus, and/or progranulin re-addition may have only affected the activity of MITF rather than its abundance and subcellular localization. We also showed that treatment with an MITF inhibitor was insufficient to significantly change the amount of GPNMB protein in both B6 and *Grn* KO pMacs within 24 hours. Further investigation into ML329 treatment showed that 24 hours of 5uM significantly decreased *Gpnmb* transcript abundance relative to vehicle control in *Grn* KO males, with smaller, insignificant effects appreciable in other groups at the same timepoint. However, 48 hours with 5uM ML329 did not significantly alter *Gpnmb* transcript in any group. To provide more context about the effect of ML329, we also measured cathepsin D transcript expression in both B6 and *Grn* KO pMacs. The vehicle controls show that *Grn* KO males have increased *Ctsd* transcript abundance relative to vehicle treated B6 male pMacs, while vehicle treated female pMacs show no significant difference regardless of genotype. 24 hours of ML329 treatment had a significant decrease in *Ctsd* transcripts in both male and female *Grn* KO pMacs with no significant change in B6 pMacs. 48 hours of ML329 treatment, however, showed differences between male and female *Grn* KO pMacs, where males continue to have decreased *Ctsd* transcript abundance, but females return to vehicle control levels. This suggests that MITF may contribute to *Gpnmb* transcription, and possibly other CLEAR genes, but is not required. This also suggests that there are other mechanisms that regulate *Gpnmb* transcription as 48 hours of the same concentration of ML329 had higher *Gpnmb* transcript levels than 24 hours of ML329 treatment. Further investigation using Cut&Run analysis of MITF-bound genes would provide deeper insight into MITF activity in pMacs in both the presence or absence of progranulin. In addition, future studies will focus on investigating involvement of other TFEB family proteins in this process.

Throughout this work, we have reported differences between genotypes, but we also demonstrate some appreciable differences with sex. This highlights one of the strengths of using pMacs differentiated within the host animal, and ongoing research is investigating functional outcomes of the GPNMB phenotype in progranulin-deficient macrophages with respect to sex. Presently, our findings represent a significant contribution to discovering additional early-stage dysregulated proteins (GPNMB and MITF) in progranulin-deficient peripheral immune cells which have known contributions to peripheral immune cell activity. GPNMB has been demonstrated to impact macrophage and T cell activity (29, 30, 42) and while the mechanism(s) are unclear, GPNMB has been previously associated with risk for PD (18, 43) and, with some controversy, found to be increased in AD patients and mouse models of AD (44–48). MITF also has significant association to immune cells. Multiple co-factors and transcription factors, including PU.1, and NFATc1, interact with MITF, influencing the differentiation of immune cells (49–51). Elucidating the consequences of the dysregulation of MITF and perhaps other TFEB family factors on the composition and integrity of progranulin-deficient peripheral immune cell populations is a novel avenue of research that will permit further insight into the role of central-peripheral crosstalk in neurodegenerative diseases.

## Data Availability

The raw data supporting the conclusions of this article will be made available by the authors, without undue reservation.
